# Cell dynamics in Hertwig's epithelial root sheath are regulated by β‐catenin activity during tooth root development

**DOI:** 10.1002/jcp.30243

**Published:** 2020-12-30

**Authors:** Siqin Yang, Hwajung Choi, Tak‐Heun Kim, Ju‐Kyung Jeong, Yudong Liu, Hidemitsu Harada, Eui‐Sic Cho

**Affiliations:** ^1^ Cluster for Craniofacial Development and Regeneration Research, Institute of Oral Biosciences Jeonbuk National University School of Dentistry Jeonju Republic of Korea; ^2^ Division of Developmental Biology and Regenerative Medicine, Department of Anatomy Iwate Medical University Shiwa‐gun Japan

**Keywords:** cell junction, epithelial‐to‐mesenchymal transition, Hertwig's epithelial root sheath, tooth root, β‐catenin

## Abstract

β‐catenin, a key mediator of Wnt signaling, plays multiple roles in tooth development. However, the role of β‐catenin in Hertwig's epithelial root sheath (HERS) during root formation remains unclear. In this study, we generated inducible tissue‐specific *β‐catenin* conditional knockout mice (*Ctnnb1* ^i∆shh^) to investigate how β‐catenin in HERS affects tooth root development. The inactivation of *β‐catenin* in HERS led to interrupted root elongation due to premature disruption of HERS. This phenotype was accompanied by reduced cell‐cell adhesion and decreased expression of junctional proteins, as well as increased epithelial‐to‐mesenchymal transition of HERS cells upon β‐catenin depletion. Accordingly, stabilization of *β‐catenin* in HERS (*Catnb* ^i∆shh^) led to the formation of unfragmented HERS and resulted in the failure of HERS dissociation, with increased expression of junctional proteins. Our results suggest that fine control of β‐catenin is important for HERS to guide root formation through regulating its structural integrity.

## Graphical abstract

We demonstrated that tooth root elongation was impaired by premature disruption of Hertwig's epithelial root sheath (HERS) following the inducible inactivation of ß‐catenin in HERS, and that HERS disruption was failed following the induction of ß‐catenin stabilization in HERS. Our results suggest that fine control of ß‐catenin is important for HERS to guide root formation through regulating its structural integrity.

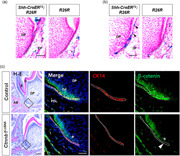

## 1 INTRODUCTION

A series of reciprocal interactions between epithelial and mesenchymal cells is important for tooth development. After the formation of tooth crown, the dental epithelium leads root formation. Around postnatal Day 4 (P4) in mice, the inner and outer enamel epithelia fuse at the lower edge of the enamel organ and form a bilayer tissue called Hertwig's epithelial root sheath (HERS; Huang et al., 2009). HERS then starts to move through invagination between the dental papilla and dental follicle until the completion of root elongation. HERS guides root formation by initiating odontoblasts differentiation from the mesenchymal cells in the dental papilla (Huang et al., 2009; Li et al., 2017). HERS functions as a signaling center to guide root formation and is considered to play major roles in determining the structure, shape, number, length, and size of tooth root through the epithelial–mesenchymal interactions involved in all steps of tooth development (Huang et al., 2009; Huang et al., 2010; Li et al., 2017; Luder, 2015; Thomas, 1995).

Previous researchers analyzed the dynamics of HERS during further root development by examining its immunoreactivity for keratin and using a transgenic reporter system for the *Keratin‐14* (*K14*) gene in mice (Huang et al., 2009; Luan et al., 2006). Those studies imply that root dentin apposition may cue HERS for dissociation. Shortly after the deposition of mantle dentin matrix layer, around P10 in mice, HERS begins to disintegrate (Huang et al., 2009), though it still undergoes invagination at the apex. HERS remains in a network structure over the root dentin surface, which results in the formation of the epithelial cell rests of Malassez (ERM; Luan et al., 2006; Xiong et al., 2013). Although the formation of HERS during initial root development has been studied extensively, questions about the molecular network that controls the cell dynamics and the fate of HERS cells during further tooth root development remain unanswered: how is the dissociation of HERS initiated? How is the epithelial‐to‐mesenchymal transition (EMT) of HERS cells initiated and incorporated into the formation of periodontal tissues?

The canonical Wnt signaling pathway involves the stabilization and nuclear accumulation of β‐catenin, which activates the expression of Wnt target genes (Clevers, 2006). The Wnt/β‐catenin signaling plays multiple roles in both the dental epithelium and dental mesenchyme during various stages of tooth development (F. Liu & Millar, 2010). Constitutive stabilization of *β‐catenin* in the dental epithelium leads to continuous ectopic tooth formation in embryos (Jarvinen et al., 2006; F. Liu et al., 2008), whereas inactivation of *β‐catenin* in the dental mesenchyme arrests tooth development at the bud stage (Chen et al., 2009). During crown formation, epithelial β‐catenin depletion leads to variable enamel defects caused by improper ameloblast differentiation (Guan et al., 2016; Yang et al., 2013). In addition, Wnt/β‐catenin signaling is essential for root formation, as demonstrated using a mouse model with inactivation of *β‐catenin* in the dental mesenchyme (Kim et al., 2013). Recently, targeting the dental epithelium using a *K14* promoter has been applied in studying root development (Huang et al., 2010; Li et al., 2015). However, even using that system, it can be difficult to observe postnatal root development and determine whether a root defect is secondary to a crown defect.

To investigate the role of β‐catenin in the cell dynamics of HERS and determine the significance of Wnt/β‐catenin signaling in HERS during root formation, we analyzed mice with tamoxifen‐induced modulation of β‐catenin in their HERS in this study. To understand the cellular and molecular mechanisms underlie HERS dynamics, we also modulated the level of β‐catenin in immortalized HERS cells. Taken together, our results reveal that β‐catenin is important for the cell dynamics and functions of HERS that eventually regulate tooth root development.

## 2 MATERIALS AND METHODS

### 2.1 Mouse strains

All procedures were performed in accordance with the National Institutes of Health Guidelines on the Use of Laboratory Animals. All experimental procedures were approved by the Animal Welfare Committee of Jeonbuk National University. *β*‐*catenin* floxed allele (*Ctnnb1* ^CO/CO^), *ShhcreER* ^T2^, and *R26R* reporter mice (Brault et al., 2001; Harfe et al., 2004; Soriano, 1999) were purchased from Jackson Laboratory (Bar Harbor). To generate inducible inactivation or stabilization alleles of the β‐catenin gene in HERS cells, *ShhcreER* ^T2^ allele mice were crossed with *Ctnnb1* ^CO/CO^ or *Catnb* ^lox(ex3)/+^ mice, respectively (Brault et al., 2001; Harada et al., 1999). The genotypes of the mouse offspring were determined as previously described (Brault et al., 2001; Harada et al., 1999; Harfe et al., 2004).

### 2.2 Tamoxifen administration and *X‐gal* staining

Tamoxifen (Sigma‐Aldrich) was dissolved in corn oil (Sigma‐Aldrich) at 20 mg/ml. To induce Cre‐recombination in *ShhcreER* ^T2^:*Ctnnb1* ^CO/CO^ (*Ctnnb1* ^i∆shh^) and *ShhcreER* ^T2^:*Catnb* ^lox(ex3)/+^ (Catnb^i∆shh^) mice, tamoxifen was injected intraperitoneally with a single injection at 150 mg/kg body weight. Age‐matched *Ctnnb1* ^CO/CO^ or *Catnb* ^lox(ex3)/+^ mice were used as the control. At least five independent littermates were used for each experimental group (*n* ≥ 5/genotype, including males and females, age indicated in the figure). To analyze the Cre activity induced in *ShhcreER* ^T2^ mice, *ShhcreER* ^T2^ mice were crossed with *R26R* mice, and tamoxifen was injected intraperitoneally to modulate β‐catenin in HERS at the indicated stage. Then, using a previously described method (Kim et al., 2013), *X‐gal* staining was performed with the jaws of *ShhcreER* ^T2^:*R26R* and *R26R* mice.

### 2.3 Tissue preparation, histology, and histomorphometry

The mice were killed, and their mandibles were carefully dissected for histological analysis. Tissues were fixed in 4% paraformaldehyde (Sigma‐Aldrich) and decalcified in 10% EDTA for 2 to 4 weeks at 4°C. The decalcified tissues were dehydrated through a graded ethanol series, embedded in paraffin, and sectioned at 5‐μm using standard histological procedures as formerly described (Kim et al., 2013). Slides were stained with hematoxylin and eosin (H–E). The root length was measured with H‐E‐stained frontal sections of the distal root of the mandibular first molars using the AnalySIS Pro imaging system (Soft Imaging System) and was determined as the distance from the cemento‐enamel junction to the apical end of the root dentin. The measurements were performed on three representative slides from each group.

### 2.4 Immunohistochemistry and immunofluorescence staining

For immunostaining, sections were treated with 3% hydrogen peroxide and incubated with rabbit polyclonal antibodies against β‐catenin (RB‐9035; Thermo Fisher Scientific), cytokeratin 14 (CK14; ab7800; Abcam), E‐cadherin (E‐Cad; sc‐7870; Santa Cruz Biotechnology), laminin (ab11575, Abcam), tissue nonspecific alkaline phosphatase (Tnap; 11187‐1‐AP; Proteintech), osterix (Osx; ab22552; Abcam), and nestin (MAB353; Chemicon International). A rabbit‐specific HRP/DAB detection IHC kit (Abcam) was used according to the manufacturer's instructions. For immunofluorescence staining, tissue sections were permeabilized by incubation in 0.2% Triton X‐100 for 10 min at room temperature. After rinsing with phosphate‐buffered saline (PBS), nonspecific binding sites on the cells were blocked with 5% bovine serum albumin in PBS for 30 min at room temperature. Tissue sections were then incubated with primary antibodies against β‐catenin and CK14 (ab7800; Abcam) for 16 h at 4°C. Normal rabbit or mouse immunoglobulin (Santa Cruz Biotechnology) was used as a negative control for the primary antibodies. Alexa Fluor®‐conjugated secondary antibodies (Invitrogen) were used for detection. DAPI was used for counterstaining. Cell staining was evaluated using a fluorescence microscope (Leica Microsystems).

### 2.5 Cell culture, transfection, and stable cell lines with short hairpin RNA

HERS01a, a mouse HERS cell line, was cultured as described previously (Akimoto et al., 2011). MDPC‐23 cells (Hanks et al., 1998), a murine dental papilla cell line, were maintained in Dulbecco's modified Eagle medium (Invitrogen) with 10% fetal bovine serum (Invitrogen), and 100 IU/ml penicillin‐100 μg/ml streptomycin (Invitrogen). To generate retroviral particles, small hairpin RNA (shRNA) against mouse *Ctnnb1* (TG500280) and control shRNA (TR30013) were purchased from OriGene Technologies (Rockville). The establishment of stable cell lines by viral transductions were conducted as previously described (Choi et al., 2017). After establishment of stable cell lines with shRNA, conditional media were harvested from HERS cells, mixed 50:50 with the growth medium for MDPC‐23 cells, and then administered to MDPC‐23 cells for 24 h. The plasmid driving the expression of mouse *β‐catenin S33Y* was a gift from Shinya Yamanaka (Addgene plasmids #13371). DNA constructs were transfected by using Lipofectamine^TM^ LTX and PLUS reagent (Invitrogen) according to the manufacturer's instructions.

### 2.6 Statistical analysis and supporting information

Data are presented as mean ± standard error of the mean of three or more separate experiments. Normal data with equal variance were analyzed using Student's *t* test and *p* < .05 was considered statistically significant. Detailed descriptions for other experimental materials and methods are provided in the Supporting Information.

## 3 RESULTS

### 3.1 Targeting HERS to modulate β‐catenin activity during root formation

**Figure 1:**
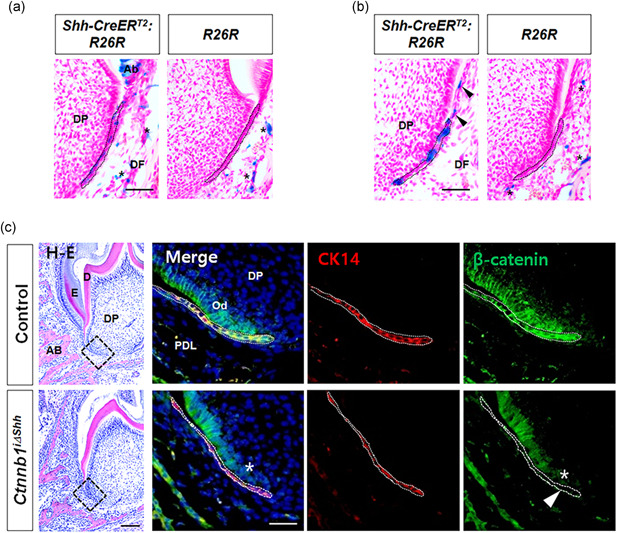
Targeting HERS to modulate β‐catenin activity during root formation. (a) LacZ activity in Hertwig's epithelial root sheath (HERS) cells undergoing invagination was analyzed by *X‐gal* staining of the mandibular molars of *Shh‐CreER* ^T2^ *:R26R* double transgenic mice at P8 following tamoxifen administration at P4. HERS is indicated with dotted black lines. The asterisks are the endogenous background, as displayed in the control *R26R* mice. Scale bars = 20 μm. (b) LacZ activity in HERS cells undergoing invagination and dissociation was analyzed by *X‐gal* staining at P12 following tamoxifen administration at P8. Black arrows indicate Shh‐expressing cells dissociated from HERS. (c) After tamoxifen administration at P4 to induce Cre activity in HERS, tissue sections of the mesial root of the first molar from control and *Ctnnb1* ^i∆shh^ mice at P10 were stained with H–E (left) and immunofluorescence (right). Dotted black squares in the H–E‐stained images indicate the area of HERS co‐stained with immunofluorescences for CK14 and β‐catenin. HERS is indicated with dotted white lines. The white arrowhead indicates that the HERS of *Ctnnb1* ^i∆shh^ mice positive for CK14 show reduced expression of β‐catenin compared with the control, which clearly co‐expresses CK14 and β‐catenin in HERS. The white asterisk indicates reduced expression of β‐catenin in the dental papilla cells contacting the HERS of the *Ctnnb1* ^i∆shh^ mice. Scale bars = 100 μm (H–E), 10 μm (immunofluorescence). AB, alveolar bone; Ab, ameloblast; D, dentin; DF, dental follicle; DP, dental papilla; E, enamel; Od, odontoblast; PDL, periodontal ligament

During tooth development, Shh is primarily expressed in the dental epithelium, including HERS, from initiation to the root formation stages (Hosoya et al., 2020; Y. Liu et al., 2015). Following a previous systemic analysis of postnatal root development (Y. Liu et al., 2015), we used the tamoxifen‐induced *ShhcreER* ^T2^ */loxP* system in mice. Through the LacZ reporter expression of *ShhcreER* ^T2^ *:R26R* double transgenic mice after tamoxifen administration, we traced the effects of tamoxifen to confirm that recombination occurred. *X‐gal* staining for LacZ activity revealed that Shh expression was restricted in the developing dental epithelium, including HERS under invagination and dissociation, after tamoxifen administration to mice aged P8 and P12 (Figure 1a,b). The *X‐gal*‐positive HERS was not continuous with interruptions at either stage, suggesting a mosaic pattern of Shh expression in HERS. In the generation of mice obtained by crossing a *β*‐*catenin* floxed allele with *ShhcreER* ^T2^ mouse, the inactivation of *β*‐*catenin* in HERS (*Ctnnb1* ^i∆shh^) was induced by tamoxifen administration at P4 and analyzed at P10 and P14 to determine the dynamics and fate of HERS during root formation. The subsequent *β*‐*catenin* inactivation in HERS was confirmed using immunostaining of dental tissues at P10 (Figure 1c). The HERS cells were positive for CK14, which is an epithelial cell marker, at the apex of the growing tooth root. In the control mice, *β*‐*catenin* was highly expressed in both HERS and the odontoblast. However, the *Ctnnb1* ^i∆shh^ mutant had reduced β‐catenin expression in the CK14‐positve HERS and lower than normal expression in preodontoblasts and dental papilla cells contacting HERS, suggesting that β‐catenin in HERS also regulates the gene expression of β‐catenin in neighboring preodontoblasts and dental papilla cells through epithelial‐mesenchymal interactions.

### 3.2 Inactivation of β‐catenin in HERS disrupts the bilayer structure of HERS

**Figure 2:**
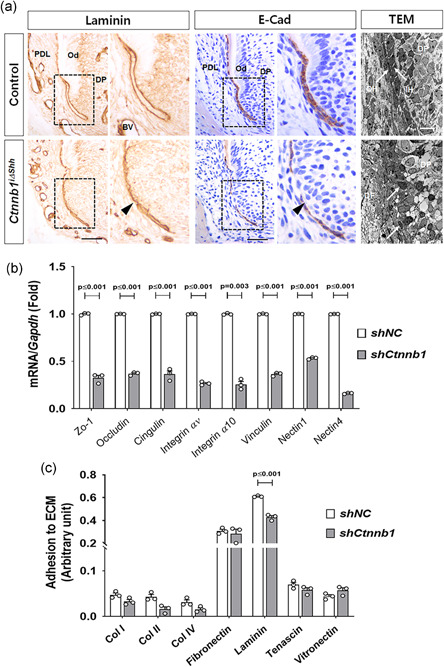
Disrupted structure of the bilayer HERS by inactivation of β‐catenin. (a) The morphological and structural differences between the HERS of *Ctnnb1* ^i∆shh^ and control mice are shown using the immunolocalization of laminin and E‐Cad and transmission electron microscope (TEM) images of tissue sections from the mandibular first molar at P10 after tamoxifen administration at P4. The area of the dotted black squares of laminin and E‐Cad is magnified at the right side of the images. The black arrowheads indicate the thinner dissociated HERS of the *Ctnnb1* ^i∆shh^ mice compared with the control. The TEM image of HERS in the *Ctnnb1* ^i∆shh^ mice was compared with the control at P10. The white arrows indicate double layered HERS cells with inner (IH) and outer layers (OH) in the control and cells from the disrupted HERS in the *Ctnnb1* ^i∆shh^ mice. Scale bars = 20 μm (immunohistochemistry [IHC]), 10 μm (TEM). (b) Messenger RNA (mRNA) transcript levels were analyzed by real‐time quantitative polymerase chain reaction (qPCR). RNA was isolated from HERS01a cells transduced with retroviruses harboring short hairpin RNA (shRNA) for *Ctnnb1* (*shCtnnb1*) or the negative control (*shNC*). (c) The adhesion ability of the cells to various kinds of ECM was analyzed and compared. Significance was assigned for *p* values as indicated. BV, blood vessel; DF, dental follicle; DP, dental papilla; ECm, extracellular matrix; HERS, Hertwig's epithelial root sheath; IH, inner layer of HERS; OH, outer layer of HERS; Od, odontoblast; PDL, periodontal ligament

HERS development begins with the formation of a bilayer extension of the inner and outer dental epithelium from the cervical loop of the enamel organ. The bilayer morphology is a hallmark of HERS and thought to be important for the function of movement and potentially for epithelial‐mesenchymal interactions during root development (Sakano et al., 2013). The immunolocalization of laminin has been used to display the epithelial‐mesenchymal junction in the basement membrane of the tooth germ (Mullen et al., 1999; Thesleff et al., 1981). We used a pan‐specific laminin antibody that reacts with all isoforms of laminin to analyze the outline structure of the HERS and ERMs of mice at P10. The immunolocalization of laminin clearly showed a difference in the structure of HERS; the thickness of HERS in *Ctnnb1* ^i∆shh^ mice was thinner than in the control mice, whose HERS had a double layers, suggesting that the inactivation of *β‐catenin* in HERS cells disrupts the bilayer HERS structure (Figure 2a). More interestingly, as shown by the immunolocalization of E‐Cad, which is another marker of epithelium, including HERS (Sonoyama et al., 2007), the cells in the HERS of *Ctnnb1* ^i∆shh^ mice were partially dissociated on P10 (Figure 2a). That represents a premature dissociation of HERS because HERS dissociation usually occurs on the surface of the root dentin in the process of ERM generation following dentin apposition during root elongation (Huang et al., 2009). An ultrastructural analysis using a transmission electron microscope (TEM) confirmed the disrupted structure of HERS in *Ctnnb1* ^i∆shh^ mice compared with the control at P10 (Figure 2a). Particularly, β‐catenin is a key component of Wnt signaling pathway and also functions as a component of the cadherin complex that controls cell‐cell adhesion and influences cell migration (Nelson & Nusse, 2004). To investigate how β‐catenin regulates the HERS structure, we used HERS01a cells, a HERS cell line established with mouse molar germs that has a well verified expression profile (Akimoto et al., 2011). HERS01a cells with stable expression of shRNA specific for β‐catenin (*shCtnnb1*) were verified by comparing the protein level of β‐catenin (Figure S1a) and messenger RNA (mRNA) levels of its transcriptional targets (*Lef1* and *Axin2*) with those from cells transfected with the negative control shRNA (*shNC*; Figure S1b). Interestingly, the *Ctnnb1*‐knockdown HERS01a cells (*shCtnnb1*) exhibited lower cell‐cell contact during cell culture than the control (*shNC*; Figure S1c). As expected, HERS01a cells with *shCtnnb1* exhibited significantly lower mRNA expression levels of junctional proteins, including *zonula occludens‐1*, *occludin*, *cingulin*, *integrin αν*, *integrin α10*, *vinculin*, *nectin1*, and *nectin4*, compared to the control (Figure 2b). Next, to evaluate the adhesion ability of the cells to various kinds of extracellular matrix, including collagen 1, collagen 2, collagen 4, fibronectin, laminin, tenascin, and vitronectin, adhesion array was performed. It showed that HERS01a cells bind strongly to laminin and fibronectin and that *Ctnnb1*‐knockdown HERS01a cells show significantly lower adhesion to laminin than the control. These results thus suggest the functional influence of β‐catenin in regulating the structural integrity of HERS as a component of junctional complexes (Figure 2c).

### 3.3 Increased epithelial‐to‐mesenchymal transition of HERS cells by inactivation of β‐catenin

**Figure 3:**
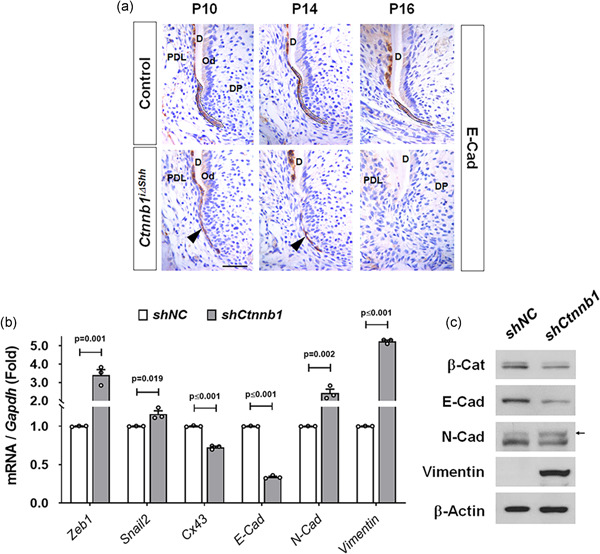
Increased epithelial‐mesenchymal transition (EMT) of HERS cells by inactivation of β‐catenin. (a) Chronological changes in morphology and molecular changes in the HERS of *Ctnnb1* ^i∆shh^ and control mice were compared using E‐Cad‐stained tissue sections of the mesial root of the first molar at the indicated ages after tamoxifen administration at P4. The intact HERS of the control is indicated with dotted black lines. The black arrowheads indicate the thinner and dissociated HERS of the *Ctnnb1* ^i∆shh^ mice compared with those of the control. Scale bar = 20 μm. (b) mRNA transcript levels of EMT‐associated genes were analyzed by real‐time qPCR. RNA was isolated from HERS01a cells transduced with retroviruses harboring shRNA for *Ctnnb1* (*shCtnnb1*) or the negative control (*shNC*). Significance was assigned for *p* values as indicated. (c) The protein levels were analyzed by western blot analysis. The samples were derived from the same experiment, and gels/blots were processed under the same experimental conditions. β‐Actin was used as a loading control. β‐Cat, β‐catenin. D, dentin; DP, dental papilla; HERS, Hertwig's epithelial root sheath; mRNA, messenger RNA; Od, odontoblast; PDL, periodontal ligament; qPCR, quantitative poltmerase chain reaction; shRNA, short hairpin RNA

To trace the fate of the HERS disrupted by the inactivation of *β‐catenin*, we analyzed the morphologies of HERS chronologically using the immunolocalization of E‐Cad as long as the *Ctnnb1* ^i∆shh^ mice could survive. Unfortunately, all the *Ctnnb1* ^i∆shh^ mice died around P16 before the full development of their tooth roots. We found that during root development, the E‐Cad‐positive HERS of *Ctnnb1* ^i∆shh^ mice was single‐layered and extended toward the apex at P10, began to disappear from the middle portion of HERS at P14, and disappeared completely at P16, whereas the bilayer HERS of the control mice was bent inward at P10, continued to move in apical direction for root elongation at P14, and remained intact at the most apical portion at P16 (Figure 3a). To address how the E‐Cad‐positive HERS cells were reduced in the tooth root apex of *Ctnnb1* ^i∆shh^ mice, we traced the apoptotic fate of HERS using in situ cell death detection assay. However, apoptosis was undetectable in the HERS of *Ctnnb1* ^i∆shh^ mice (Figure S2a). We also analyzed the proliferation status of HERS cells using the immunoreactivity of Ki67. Few proliferating cells were detected in the HERS of *Ctnnb1* ^i∆shh^ mice at P10, whereas the cells apically located in the HERS of the controls were strongly positive for Ki67 (Figure S2b). The lower proliferation rate of HERS cells with *β‐catenin* inactivation was also confirmed using an in vitro proliferation assay comparing *Ctnnb1*‐knockdown HERS01a cells (*shCtnnb1*) with the control (*shNC*; Figure S2c). We used quantitative real‐time PCR to examine the expression of EMT‐associated molecules and found that compared with the control, *Ctnnb1*‐knockdown HERS01a cells retained significantly higher expression levels of EMT markers, such as *Zeb1* and *Snail2*, lower expression of *E‐Cad* and *connexin 43* (*Cx43*), which are epithelial and junctional proteins, respectively, and higher expression of *N‐cadherin* (*N‐Cad*) and *vimentin*, which are well‐known molecular markers of mesenchymal cells (Figure 3b). We also confirmed the consequent change in the protein level of the molecules following *β‐catenin* inactivation in HERS cells (Figure 3c). Therefore, we conclude that the HERS cells of *Ctnnb1* ^i∆shh^ mice initially have reduced cell‐cell adhesion ability and lower expression of junctional proteins, as well as lower cell proliferation rate than the control cells, and that eventually undergo EMT because of the inactivation of *β‐catenin*.

### 3.4 β‐catenin in HERS regulates odontogenic differentiation of dental papilla

**Figure 4:**
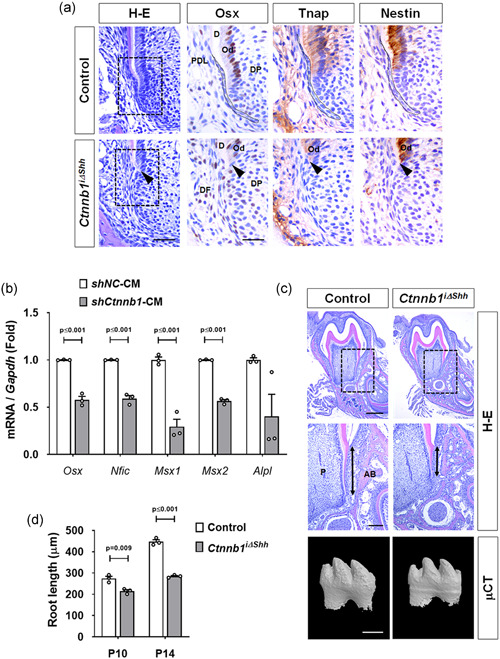
β‐Catenin in HERS regulates the odontogenic differentiation of dental papilla. (a) Morphological and molecular changes in the developing tooth roots of *Ctnnb1* ^i∆shh^ mice were detected with H–E and IHC staining. Dotted black squares in the H–E stained images indicate the magnified area of the root apex stained with IHC. Immunolocalization of Osx, Tnap, and nestin in the odontoblasts or sub‐odontoblasts was analyzed in the mandibular first molars at P14 after tamoxifen administration at P4. The intact HERS of the control is indicated with dotted black lines. The black arrowheads indicate the sudden interruption of odontoblast differentiation caused by HERS dissociation in the *Ctnnb1* ^i∆shh^ mice. Scale bars = 20 μm (H–E), 10 μm (IHC). (b) mRNA transcript levels of odontogenesis‐associated genes were analyzed by real‐time qPCR. RNA was isolated from MDPC‐23 cells treated for 24 h with conditioned medium from the culture of HERS01a cells transduced with retroviruses harboring shRNA for *Ctnnb1* (*shCtnnb1*‐CM) or the negative control (*shNC*‐CM). Significance was assigned for *p* values as indicated. (c) The difference in the root length of the *Ctnnb1* ^i∆shh^ and control mice is shown by H–E‐stained frontal tissue sections from the mandibular first molar at P14 after tamoxifen administration at P4. The dotted black squares in the upper images indicate the area of tooth root magnified below. The two‐sided black arrows indicate the difference in the root lengths of the distal root of the mandibular first molars. The bottom images are micro‐CT images of the mandibular first molar from the *Ctnnb1* ^i∆shh^ and control mice at P14 after tamoxifen administration at P4. Scale bars = 400 μm (H–E top), 100 μm (H–E bottom), 200 μm (μCT). (d) Root length was measured as the distance from the cemento‐enamel junction to the apical end of the root dentin using the distal root of the mandibular first molars from the control and *Ctnnb1* ^i∆shh^ mice at P10 and P14. Significance was assigned for *p* values as indicated. AB, alveolar bone; CT, computed tomography; D, dentin; DP, dental papilla; HERS, Hertwig's epithelial root sheath; IHC, immunohistochemistry; mRNA, messenger RNA; Od, odontoblast; P, pulp; PDL, periodontal ligament; qPCR, quantitiative polymerase chain reaction; shRNA, short hairpin RNA

During root development, HERS moves together with the dental papilla toward the apical side, followed by odontoblast differentiation to form the calcified dentin of the root (Huang et al., 2009; Li et al., 2017). Coordination between HERS and the apical papilla is crucial for proper root development (Kumakami‐Sakano et al., 2014). To further trace how premature disruption of HERS through the inactivation of *β‐catenin* affects root development, we analyzed the expression of odontogenesis‐associated proteins, including Osx, Tnap, and nestin, as markers of differentiated odontoblasts in the molars of the *Ctnnb1* ^i∆shh^ mice. In the control mice at P14, root dentin was being formed with intact HERS at the apex, and Osx‐positive odontoblasts were well‐organized inside the root dentin (Figure 4a). However, in *Ctnnb1* ^i∆shh^ mice, further development of the root dentin was interrupted, probably by the failure of odontogenesis beginning with the administration of tamoxifen (Figure 4a). Consistently, the Osx‐positive cells in *Ctnnb1* ^i∆shh^ mice were distributed in the dental follicle and papilla at the apex of the interrupted root dentin, suggesting that Osx‐positive odontogenic cells got lost and mixed with the dental follicle and papilla cells (Figure 4a). The immunoreactivity of Tnap and nestin also indicate the interrupted formation of root dentin in *Ctnnb1* ^i∆shh^ mice caused by the sudden targeting of HERS that occurred with the administration of the tamoxifen (Figure 4a). To evaluate the effect that factors released from HERS have on odontogenesis, conditional media from the cell cultures of *Ctnnb1*‐knockdown HERS01a cells (*shCtnnb1*‐CM) and control cells (*shNC*‐CM) were administered to MDPC‐23 cells. Interestingly, the MDPC‐23 cells treated with conditional medium from *Ctnnb1*‐knockdown HERS01a cells (*shCtnnb1*‐CM) exhibited significantly reduced mRNA expression levels of genes, such as *Osx*, *Nfic*, *Msx1*, and *Msx2* compared with cells treated with conditioned medium from the control cells (*shNC*‐CM; Figure 4b). Finally, the inactivation of *β‐catenin* in HERS significantly reduced the root length (the distance from the end of the enamel to the bottom of the apical dentin, as indicated in Figure 4c, *n* = 3 per group) at P10 and P14 (Figure 4d). Therefore, we conclude that the *Ctnnb1* ^i∆shh^ mice displayed the functional failure of HERS to induce odontogenic differentiation of dental papilla cells, which subsequently interrupted root dentin formation and eventually caused shorter tooth roots than found in the control.

### 3.5 Stabilization of β‐catenin in HERS results in failure of HERS dissociation

**Figure 5:**
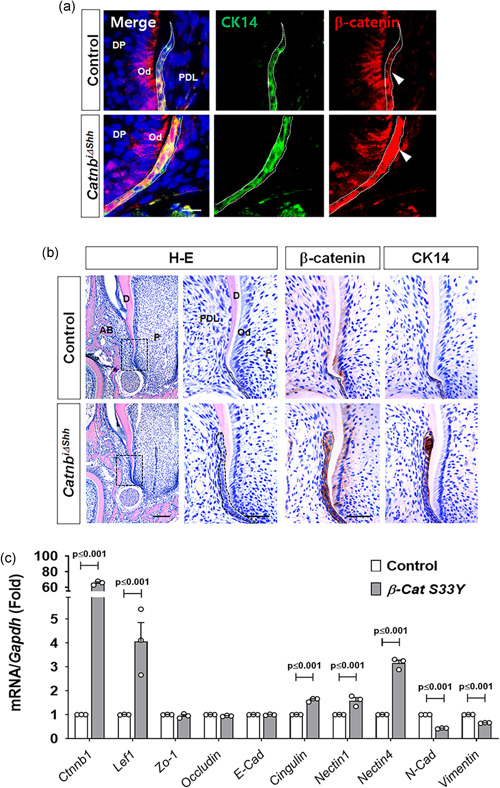
Stabilization of β‐catenin in HERS results in failure of HERS dissociation. (a) Tissue sections from the mandibular first molar in the control and *Catnb* ^i∆shh^ mice at P12 after tamoxifen administration at P8 were stained with immunofluorescence for CK14 and β‐catenin. HERS is indicated with dotted white lines. The white arrowheads indicate that the HERS of *Catnb* ^i∆shh^ mice expressing CK14 shows increased expression of β‐catenin compared with the control. Scale bars = 5 μm. (b) Morphological and molecular changes in the developing tooth roots of *Catnb* ^i∆shh^ mice were detected with H‐E and IHC staining of the mandibular first molars at P12 after tamoxifen administration at P8. The dotted black squares in the H–E stained images indicate the magnified area of root apex, including HERS. Immunolocalization of β‐catenin and CK14 in HERS was analyzed using tissue sections from the mandibular first molars at P12. The dotted black line indicates HERS. Note the thickened and undisrupted HERS of the *Catnb* ^i∆shh^ mice. Scale bars = 100 μm (H–E left), 20 μm (magnified H–E and IHC). (c) mRNA transcript levels were analyzed by real‐time qPCR. RNA was isolated from HERS01a cells transfected with a plasmid driving the expression of mouse *β‐catenin S33Y* (*β‐Cat S33Y*) for the overexpression of stable β‐catenin or the control. Significance was assigned for *p* values as indicated. AB, alveolar bone; D, dentin; DP, dental papilla; HERS, Hertwig's epithelial root sheath; IHC, immunohistochemistry; mRNA, messenger RNA; Od, odontoblast; P, pulp; PDL, periodontal ligament; qPCR, quantitiative polymerase chain reaction

Thus far, the loss‐of‐function β‐catenin mutant mice have shown the functional importance of β‐catenin in HERS for guiding root elongation by regulating its structural integrity, which is probably important for invagination and epithelial‐mesenchymal interactions during root formation. Next, we investigated the effects of a gain‐of‐function β‐catenin model in HERS using the same tamoxifen‐induced *ShhcreER* ^T2^ */loxP* system. The administration of tamoxifen at P4 to activate β‐catenin led to continuous tooth generation at the top of the crown, which indirectly affected root formation later (Figure S3). To avoid those unexpected results, we adjusted the timing of the tamoxifen to induce stabilization of *β‐catenin* in HERS to the point at which the dissociation of HERS should occur. Based on our findings from the inactivation of *β‐catenin*, we expected the activity of β‐catenin in HERS to become weaker during dissociation. After generating appropriate mice by crossing *Catnb* ^lox(ex3)/+^ and *ShhcreER* ^T2^ mice (*Catnb* ^i∆shh^), based on our results for the stage‐specific reporter expression of *ShhcreER* ^T2^ (Figure 1b), we induced the stabilization of *β‐catenin* in HERS by tamoxifen administration at P8 to test its effects on the dissociation of HERS. We confirmed that the stabilization of *β‐catenin* was limited to HERS using double staining by CK14 and *β‐catenin*‐specific antibodies with dental tissues at P12 (Figure 5a). In addition, we also checked the increased activity of β‐catenin in HERS using double staining by CK14 and Lef1‐specific antibodies (Figure S4a). In the growing mandibular molars of the *Catnb* ^i∆shh^ mice at P12, interestingly, HERS remained unfragmented and multi‐layered and extended to cover the dentin matrix, whereas HERS in the control was disrupted immediately after root dentin apposition (Figure 5b). The immunoreactivity of β‐catenin overlapped with that of CK14 within the unfragmented HERS in *Catnb* ^i∆Shh^ mice (Figure 5b). To trace the molecular change in HERS cells with the stabilization of *β‐catenin*, we transfected HERS01a cells with a constitutively active form of *β‐catenin*, *β‐Cat S33Y* (Sadot et al., 2002) for overexpression of stabilized *β‐catenin* and then confirmed by comparing the protein level of β‐catenin, non‐phosphorylated active β‐catenin, and Lef1 (Figure S4b) and mRNA levels of *Lef1* and *β‐catenin* (*Ctnnb1*; Figure 5c). Likewise, HERS01a cells expressing stabilized *β‐catenin* exhibited higher than normal mRNA expression of junctional proteins, such as *nectin4* and *cingulin* and lower than normal expression of mesenchymal marker proteins, such as *N‐Cad* and *vimentin* (Figure 5c). Taken together, our results suggest that the stabilization of *β‐catenin* in HERS changed cell–cell adhesion enough to keep it unfragmented, even after root dentin apposition.

## 4 DISCUSSION

HERS is considered to be the developmental center for root formation that induces the formation of root dentin. The cellular dynamics of HERS (growth, movement, dissociation, migration, apoptosis, and EMT) are recognized as important for determining the number, length, and shape of the root and also for the development of the periodontium, including the periodontal ligament and cementum (Li et al., 2017; Luder, 2015). However, previous reports on tooth root defects in mice have mainly targeted the dental mesenchyme, such as *OC‐Cre* (Bae et al., 2013; Gao et al., 2009; Kim et al., 2013), *Col1a1‐Cre* (Kim et al., 2012), *Sp7‐Cre* (Rakian et al., 2013), and *Gli1‐Cre* (Y. Liu et al., 2015). Although *K14‐Cre* has been used to target dental epithelium and study root development (Huang et al., 2010; Li et al., 2015), it requires a suitable timing for gene targeting or it could become difficult to observe the root phenotype due to perinatal death of the mice; it could also be difficult to determine whether the root defect is secondary to a crown defect. In addition, HERS functions as a signaling center in a network containing multiple transcription factors and growth factors that mediate tissue‐tissue interactions to guide root development (Li et al., 2017). Therefore, it is essential to study HERS signaling using inducible gene‐targeting technologies.

To understand the stage‐ and process‐specific mechanisms through which β‐catenin exerts its in vivo regulatory activity in HERS during root development, we here applied different timing of tamoxifen induction in the *ShhcreER* ^T2^ */loxP* system to inactivate and stabilize *β‐catenin*. As previously reported, *ShhcreER* ^T2^ works by administering tamoxifen in only some of the dental epithelium, including HERS, at the desired postnatal stage of tooth development (Y. Liu et al., 2015). Our results demonstrate that the inactivation of *β‐catenin* in HERS during invagination to guide root elongation leads to premature disruption of HERS and shorter roots caused by impaired odontoblast differentiation for root elongation. Upon the inactivation of *β‐catenin in vitro*, HERS cells showed a reduced cell‐cell adhesion ability and lower expression of junctional proteins, with a lower cell proliferation rate and increased EMT as possible underlying mechanisms. From the results of loss‐of‐function in HERS experiments, we propose that β‐catenin helps HERS maintain its structural integrity during its invaginating movement and induce odontoblast differentiation for root dentin formation. Subsequently, HERS begins to become dissociated or perforated. This disintegration of HERS has been thought to accompany dentin matrix accumulation during root elongation and be crucial to the formation of periodontium, such as cementum and the periodontal ligament, irrespective of whether that process is active or passive. The formation of a mesh‐like structure in HERS is thought to allow dental follicle cells to contact the newly formed root dentin surface through the perforated epithelial root sheath (Li et al., 2017; Luan et al., 2006). These dental follicle cells differentiate into cementoblasts to form cementum. We stabilized *β‐catenin* in HERS (*Catnb* ^i∆shh^) to investigate the gain‐of‐function in HERS during disintegration, in which less cell‐cell adhesion between HERS cells is expected. As anticipated, stabilized *β‐catenin* led to the failure of HERS dissociation, which is contextually similar to our results from *β‐catenin* inactivation.

In this study, we demonstrated the roles of β‐catenin in the cell‐cell adhesions of HERS during tooth development, which has not before been carefully observed. *β‐catenin* is the central player in the Wnt signaling pathway (Cadigan & Nusse, 1997), but it is also a structural adapter protein that links cadherins to the actin cytoskeleton in cell‐cell adhesion (Bienz, 2005; Johansson et al., 2019; Lien et al., 2006). The signaling function of β‐catenin is conferred by a soluble cytoplasmic pool that is highly unstable in the absence of a Wnt signal. On the other hand, β‐catenin plays a critical structural role in cadherin‐based adhesions. That adhesion function is based on a subcellular pool of β‐catenin that is membrane‐associated and stable. The functional destination of β‐catenin in Wnt signaling or cell‐cell adhesion depends on the choice of binding partners, which can be affected by other signals. Wnt signaling and cadherin‐mediated cell adhesion are intriguingly connected via *β*‐catenin (Bienz, 2005; Hinck et al., 1994; Johansson et al., 2019; Nelson & Nusse, 2004). Increased levels of β‐catenin induced by Wnt signaling leads to the saturation of β‐catenin binding to cadherin at the plasma membrane and an increase in cell–cell adhesion (Hinck et al., 1994). Conversely, inactivation of *β‐catenin* as a component of the cell adhesion complex leads to severe developmental failures (Junghans et al., 2005; Lien et al., 2006). In all steps of tooth development, many growth and transcription factors are expressed to perform crucial functions in regulating the epithelial‐mesenchymal interactions. In the physiological context of cells in tissues, our results indicate that β‐catenin regulated by epithelial–mesenchymal interaction might participate in both ways in the cell dynamics of HERS during root development. Inputs from various cell‐signaling events can affect β‐catenin function, which could be necessary to enable the finely tuned adhesive and signaling responses required for root development through HERS. As the final outcome, our results showed the functional importance of HERS in its structural integrity regulated by β‐catenin guiding the development of odontoblast from underlying dental papilla in which induce the expression of morphogenetic regulators including Osx, Nfic, and Msx.

In summary, we modulated the activity of β‐catenin in HERS during root development using inducible gene targeting technologies. Inactivation of *β‐catenin* in HERS led to premature disruption of HERS and shorter roots through impaired odontoblast differentiation for root elongation. On the other hand, stabilization of *β‐catenin* in HERS led to the failure of HERS dissociation. Our results suggest the functional influence of β‐catenin in regulating the structural integrity of HERS and postnatal root formation, implying that Wnt/β‐catenin signaling plays a crucial role in the cell dynamics of HERS during root development. These findings could provide new insight into the molecular and cellular events that underlie root formation and offer potential instruction for future tissue engineering applications.

## CONFLICT OF INTERESTS

The authors declare that there are no conflict of interests.

## AUTHOR CONTRIBUTIONS

Siqin Yang and Hwajung Choi conceived and designed the research. Tak‐Heun Kim and Siqin Yang performed the animal experiments. The histological tissue analysis and scoring were conducted by Siqin Yang, Tak‐Heun Kim, Ju‐Kyung Jeong, Yudong Liu, and Hidemitsu Harada provides HERS01a cell line. The statistical analysis and interpretation of the results were performed by Siqin Yang, Hwajung Choi, Tak‐Heun Kim, Ju‐Kyung Jeong, Yudong Liu, and Eui‐Sic Cho. The manuscript was principally written and revised by Siqin Yang, Hwajung Choi, and Eui‐Sic Cho. All the authors critically reviewed the manuscript for important intellectual content and approved the final submitted manuscript.

## Data Availability

The data sets used and/or analyzed during the current study are available from the corresponding author on reasonable request.
